# Towards More Sustainable Sports: Analyzing the Travel Behavior of Adolescent Soccer Players in Southern Norway

**DOI:** 10.3390/ijerph19159373

**Published:** 2022-07-30

**Authors:** Aron Laxdal, Bjørn Tore Johansen, Elling Bere, Bård Erlend Solstad

**Affiliations:** 1 Department of Sport Science and Physical Education, University of Agder, 4604 Kristiansand, Norway; bjorn.t.johansen@uia.no (B.T.J.); elling.bere@uia.no (E.B.); bard.e.solstad@uia.no (B.E.S.); 2 Department of Health and Inequality, Norwegian Institute of Public Health, 0213 Oslo, Norway; 3 Norwegian Research Centre for Children and Youth Sports, Norwegian School of Sport Sciences, 0863 Oslo, Norway

**Keywords:** active travel, cycling, e-bike, football, sustainability, walking

## Abstract

Mitigating climate change is a global challenge demanding effort from all sectors, and sports are no exception. While transportation is one of the key issues regarding sustainable sports, the methods by which children and adolescents get to practice have not received much attention. The objectives of this study were, therefore, to present how adolescents in Southern Norway travel to soccer practice and assess how the mode of transportation is related to sex, socioeconomic status, age, ethnicity, and distance from home to practice. Cross-sectional data were collected from 558 adolescent soccer players (398 boys and 190 girls) representing 30 different clubs from settlements of varying rurality. While most of the participants lived within cycling distance from the field of practice, the majority opted for passive modes of transportation (55% passive vs. 45% active). A logistic regression analysis found that traveling distance and age were associated with active transportation habits, while sex, ethnicity, and socioeconomic status were not. Further research is needed to examine the main barriers to active travel for this already active population, as active transport represents an opportunity for sports to become more sustainable.

## 1. Introduction

Climate change is a global challenge that demands effort from all sectors [1,2,3]. Sports are in no way exempt from responsibility as their carbon footprint is substantial, and there are many opportunities for improvement [4]. While reducing water usage, committing to less waste, and using more environmentally friendly materials would be advantageous [5,6], sport-related travel may offer the greatest opportunity for impact [7,8].

The health of the planet is essential if we are to continue to consume and participate in sports [9,10,11]. While some sports are more dependent on environmental factors than others, all active humans rely on clean air, good nutrition, and relatively stable and predictable weather condition to thrive in their chosen activity [11]. The sporting community as a whole should therefore come to terms with the need for more sustainable sporting practices.

For sports to become more sustainable, we as a society have to acknowledge that sports as an industry is often in conflict with the environment [12]. Creating conditions where participation in sports can coexist with nature in a state of mutual well-being, security, and survival will ensure that future generations can continue to enjoy the activities that we have come to love [13,14].

Even though the concept of sustainable sports has been in the spotlight for some time [13], the methods by which children and adolescents get to practice have not received much attention. The few studies on the topic to date indicate a culture of chauffeuring young athletes to organized sporting activities, contributing to increased congestion during rush hour [7,15,16,17]. While the reasons for opting for travel by car are multifactorial, previous studies have pointed to time concerns, safety concerns, distance from home to the field of practice, and the built environment as determinants [12,15,18,19]. Various sociodemographic factors such as age, ethnicity, and socioeconomic status have previously been found to be associated with active travel behavior in other contexts [16,17,20].

With organized sports being as popular as they are in Norway, with 93% of all kids participating at some point during their childhood [21], getting more athletes to opt for active modes of transport instead of motorized ones could have a meaningful environmental impact [4]. As soccer is by far the most popular sport in Norway, with 251,932 registered participants [22], it has the opportunity to become an example to other sports and utilize its social capital to become a catalyst for change.

Although most research on active transport focuses on health improvement and increasing daily physical activity [23,24], kids who participate in organized sports are already physically active. Most of them perform multiple bouts of moderate-to-vigorous physical activity a week and may therefore be open to active modes of transportation for reasons relating to sustainability and the environment as opposed to health promotion.

The objectives of this study were, therefore, (1) to present how adolescents in Southern Norway travel to soccer practice and (2) to assess how the mode of transportation is related to sex, age, ethnicity, distance from home to practice and socioeconomic status.

## 2. Methods

### 2.1. Sample and Procedure

Data were gathered from 558 soccer players between the age of 13 and 19 (398 boys, 190 girls; mean age = 15.7, SD = 1.4) from 30 soccer clubs in Southern Norway (59% of the 51 youth clubs in Agder county; all of whom were invited to participate). The minimum sample size of 526 was determined based on the studied population (youth soccer players in Southern Norway; 2274 players). The goal was to sample at least 20% of the population with a confidence level of 95% and a less than 3% margin of error. Participants were recruited in clusters, with individual teams serving as the clusters. In an effort to recruit a representative study population, teams from various types of municipalities, ranging from urban to rural, were recruited. The data collection was performed using an electronic questionnaire during the fall of 2021. The players answered the questionnaire on their personal mobile devices at the beginning or the end of a practice session after receiving a link or QR code that had been provided to the coach by a member of the research group.

The project was approved by the Norwegian Center for Research Data and the ethics committee of the Faculty of Health and Sport Sciences at the University of Agder prior to the data collection. After receiving consent from the general managers of the respective soccer clubs, the coaches of the various teams were contacted. The coaches then relayed the relevant information to the players and their legal guardians so that they could make an informed decision regarding participation in the project. Information given included background on the project, what participation would involve, and that consent could be withdrawn at any point. Players that had reached the age of 16 were allowed to consent on their own, while legal guardians consented on behalf of players under the age of 16.

### 2.2. Measures

Mode of transport to soccer practice was measured using a multiple-choice question asking, “which mode of transportation do you usually take to practice?”. Response options were by foot, by bike, by e-bike, by e-scooter, on a bus, in a car, and by motorbike. An open-ended question asking, “how far from the practice field do you live?” assessed the distance from the player’s home to the practice field.

Socioeconomic status was measured using the Family Affluence Scale (FAS III [25]), which consists of six questions that approximate family affluence. The measure has been found to be a valid and reliable way to measure information that adolescents are sometimes unwilling or unable to reveal [26]. Examples of items are “Does your family own a car or a truck?” (response categories were “No”, “Yes, one”, and “Yes, two or more”) and “Do you have your own bedroom for yourself?” (response categories were “No” and “Yes”). A dichotomous composite score was calculated, grouping the respondents into high or low socioeconomic status.

Other variables of interest were age, sex, and ethnicity, where ethnicity was grouped into native and immigrant backgrounds depending on the birthplace of the player, their parents, and their grandparents.

### 2.3. Statistical Analysis

A logistic regression analysis was used to assess the relationship between the independent variables (i.e., age, sex, ethnicity, traveling distance, and socioeconomic status) and active transport. The non-linear analysis produced an odds ratio and 95% confidence intervals for all independent variables. All statistical analyses were performed in SPSS (version 28.0; IBM Corp., Armonk, NY, USA), with significance accepted as long as the 95% confidence interval of the odds ratio did not include 1.

## 3. Results

As can be seen in Table 1, the majority of adolescents opted for passive transportation to practice (55% passive- vs. 45% active transport), with cars being the most common mode of transportation (39%). The median distance to training was 3.0 km (Q2 = 1.5, Q3 = 5.0), with most of the participants living within cycling distance (27% within 2 km, 67% within 4 km, and 91% within 10 km). The popularity of the bicycle appears to decrease with age, corresponding with the legal age for driving (16 for motorbikes and 18 for automobiles).

A logistic regression analysis found the odds of opting for passive transport to practice to increase with traveling distance and age (see Table 2). Sex, ethnicity, and socioeconomic status were not significantly associated with mode of transportation from home to practice. The overall (Nagelkerke) R^2^ was 0.419.

## 4. Discussion

Despite most of the participants living within cycling distance from the field of practice, our study found that the majority of adolescent soccer players from Southern Norway opted for passive modes of transport to practice. These results are in line with the findings of previous studies, which revealed a culture of chauffeuring children to organized leisure activities in Norway [15,16]. The findings are also congruent with findings from the United States of America, where a group of swimmers were found to spend an average of 106 min a week driving the 81 cumulative kilometers to and from practice; a significant portion of the total hours spent driving each week [7].

These results run somewhat counter to findings from Spain, where walking to leisure activities appears to be much more common [17]. Whether this discrepancy is related to weather conditions, distance from the home to practice, or cultural trends is difficult to determine due to insufficient data, but the divergence between the two contexts is quite striking. The popularity of the bicycle in our sample is in line with previous studies on active transport from the Norwegian context [27], where the bike seems to be quite popular. However, the use of bicycles decreased drastically with age. This reduction is not illogical, seeing as the average distance to practice increases with age (from 3.5–7.9 km), and the players become eligible for their light motorbike- and driving licenses at 16 and 18 years old, respectively. Interestingly, the use of electric bicycles was quite substantial among the 13–16-year-olds. The electric bicycle is a healthier and more sustainable alternative to other motorized options [28,29].

These findings prompt two questions: “how far do we expect the players to travel actively?” and “why does the distance to the field of practice increase so drastically as the players age?” While the answers to these questions are beyond the scope of this paper, some preliminary reflections will be offered. As several previous studies have pointed out, organized leisure activities are often outside of the adolescents’ immediate environment [7,12,15]; however, that does not apply to most of the participants in the current study. Seeing as the median distance for the participants was only 3 km, active travel should be unproblematic for most of them. For those who reside further from the practice field and possess the required means, e-bikes would represent a mode of transport that would greatly expand the expected distance one would be expected to travel actively.

While the reasons why the traveling distance to practice increases can be many, they may be related to changes in the sport-specific needs of the players. Some might change clubs in search of the best available sporting environment, and others may opt for more suitable social environments [30]. Some might simply have to change teams as their local club no longer serves their age group. There is also the possibility that the players are still playing at the same club but that they are being made to travel further so that the younger players at the club can travel shorter distances.

Contrary to findings from the school context [20,24,31], sex, ethnicity, and socioeconomic status were not found to be associated with choosing active transport. That is not to say that there were no variations in transportation behavior across these variables, as soccer players with an immigrant background were more likely to use the bus while players of native origins were more likely to use motorbikes. Girls seem to be fonder of the e-bike than boys. As sports participation during adolescence tends to be positively correlated with socioeconomic status, many of the least affluent players will have dropped out at this point, explaining the discrepancy between the different contexts [32]. The stratified data were not analyzed further due to limited statistical power.

In line with previous recommendations for environmentally friendly mobility in sport [7,8,12,18], we propose that municipalities and sports clubs (1) build safe walking- and cycle path networks that are linked with sporting facilities, (2) schedule practices at fields that do not require the players to travel further than necessary and in timeslots that do not encourage driving, (3) increase awareness and promote a favorable attitude towards more sustainable modes of transportation, (4) encourage those who drive to practice to carpool, (5) promote or subsidize the use of e-bikes instead of motorbikes, and (6) organize or facilitate walking and cycling groups for those living in the same area.

Implementing these recommendations would likely reduce traffic and lead to more active transportation behavior among current and former soccer players. Policymakers and sporting authorities cannot continue to ignore the negative environmental outcomes of sports participation, which offsets, in many ways, the positive impact of physical activity [4]. A concerted effort to increase active transport could potentially represent a powerful contribution to the fight against climate change, and being able to practice in your immediate neighborhood would favor sustainability, thus making motorized transportation redundant [8].

Some limitations to this study should be noted. The study design does not allow seasonal variation to be taken into account. Some participants may alter their travel behavior depending on weather conditions and the time of year, and the timing of the data collection could therefore affect the results. However, collecting the data in the fall, when weather conditions for active transport are optimal, reduces the likelihood of underestimating active modes of transport substantially. The sampling procedure yielded multiple small clusters of participants that were not adequately large to perform multilevel analyses [33]. Nevertheless, this study provides important preliminary information that can inform future research that will be able to take between-cluster and within-cluster effects into consideration.

While the number of participants with an immigrant background was relatively modest (66 participants; 11%) and not representative of the Norwegian population as a whole (18.9% of inhabitants have an immigrant background [34]), it is representative of the share of sport participants with an immigrant background, which is significantly lower than the population as a whole [35]. Although participation rates were relatively modest, the participating teams represent cities, towns, and burrows that vary when it comes to rurality, socioeconomic background, and other demographics. Additional factors that could have provided a more nuanced understanding of travel behavior were not included in the study.

Future research should examine why travel distance increases so drastically with age and whether the increase is mostly due to club changes or whether other mechanisms are at play. Future research should also address whether and how distance and sustainability factor in when young athletes change clubs or whether their choices are purely based on sporting- or social reasons. Whether sustainability and transport behavior factor in during the planning phase and when municipalities and sports clubs distribute practice slots would also be of interest.

## 5. Conclusions

A minority of adolescent soccer players in Southern Norway reported active modes of transportation to soccer practice. Furthermore, traveling distance and age were found to be associated with active transportation habits, while sex, ethnicity, and socioeconomic status were not. While the reasons this already active population opts for passive modes of transportation to such an extent are unclear, these results should act as a wake-up call for the stakeholders in Norwegian youth sports. As recognized by the United Nations Framework Convention on Climate Change [36], sports have an enormous cultural impact and a unique ability to transcend barriers that others strive to overcome. That impact should not only be used for monetary gain but also to enact social change for the good of all humankind; perhaps the encouragement and normalization of active transportation is a good place to start.

## Figures and Tables

**Table 1:** Descriptive statistics for all relevant variables.

		*N*	%	Distance (km)		Mode of Transport (%)							Passive/Active (%)	
				Mean	Median	Walk	Bike	E-Bike	E-Scooter	Bus	Car	Motorbike	Passive	Active
All		588	100	4.9	3.0	8	29	9	2	3	39	11	55	45
Sex	Boys	398	68	4.9	3.0	9	30	5	2	4	39	11	56	44
	Girls	190	32	5.0	3.0	5	26	16	3	2	37	10	52	48
Age (years)	13–14	140	24	3.5	2.0	8	41	13	4		34		38	62
	15	149	25	4.7	3.0	6	33	9	5	2	45		51	49
	16	118	20	4.1	3.0	8	25	14		4	30	19	53	47
	17	118	20	6.1	3.0	9	18	4	1	7	32	31	70	30
	18–19	63	11	7.9	5.0	10	16		2	3	64	5	74	26
Ethnicity	Native	522	89	4.6	3.0	7	29	10	3	2	39	12	55	45
	Immigrant background	66	11	7.1	3.0	14	29	2	2	14	40	0	56	44
Travel distance	Less than 2 km	150	27	0.8	1.0	23	51	9	1	1	9	6	17	83
	2 to 6.9 km	298	54	3.4	3.0	2	26	12	3	2	42	13	60	40
	7 km or more	102	19	15.0	10.0	1	3	1	2	10	74	10	95	5
Socioeconomic status	Low	275	47	4.8	3.0	10	28	7	2	5	40	9	55	45
	High	313	53	5.0	3.0	6	29	11	3	1	38	12	54	46

**Table 2:** Results from the multinomial logistic regression analysis.

Variable		Passive- vs. Active Travel		
		Odds Ratio	Confidence Interval	
Sex	Girls vs. Boys	0.9	0.6	1.5
Age	15 vs. 13–14	1.7	1.0	3.1
	16 vs. 13–14	1.8	1.0	3.5
	**17 vs. 13–14**	**3.3**	**1.7**	**6.4**
	**18–19 vs. 13–14**	**4.9**	**2.1**	**11.8**
Ethnicity	Immigrant vs. native	1.0	0.5	2.1
Travel distance	**2–6.9 km vs. <2 km**	**8.2**	**4.9**	**13.6**
	**7 km or more vs. <2 km**	**94.3**	**34.3**	**259.9**
Socioeconomic status	High vs. Low	0.9	0.6	1.4

Statistically significant differences are highlighted in bold.

## Data Availability

Data are available upon request.
